# Early childhood height-adjusted total kidney volume as a risk marker of kidney survival in ARPKD

**DOI:** 10.1038/s41598-021-00523-z

**Published:** 2021-11-04

**Authors:** Kathrin Burgmaier, Samuel Kilian, Klaus Arbeiter, Bahriye Atmis, Anja Büscher, Ute Derichs, Ismail Dursun, Ali Duzova, Loai Akram Eid, Matthias Galiano, Michaela Gessner, Ibrahim Gokce, Karsten Haeffner, Nakysa Hooman, Augustina Jankauskiene, Friederike Körber, Germana Longo, Laura Massella, Djalila Mekahli, Gordana Miloševski-Lomić, Hulya Nalcacioglu, Rina Rus, Rukshana Shroff, Stella Stabouli, Lutz T. Weber, Simone Wygoda, Alev Yilmaz, Katarzyna Zachwieja, Ilona Zagozdzon, Jörg Dötsch, Franz Schaefer, Max Christoph Liebau, The ARegPKD Consortium, Kathrin Burgmaier, Samuel Kilian, Klaus Arbeiter, Bahriye Atmis, Anja Büscher, Ute Derichs, Ismail Dursun, Ali Duzova, Loai Akram Eid, Matthias Galiano, Michaela Gessner, Ibrahim Gokce, Karsten Haeffner, Nakysa Hooman, Augustina Jankauskiene, Germana Longo, Laura Massella, Djalila Mekahli, Gordana Miloševski-Lomić, Hulya Nalcacioglu, Rina Rus, Rukshana Shroff, Stella Stabouli, Lutz T. Weber, Simone Wygoda, Alev Yilmaz, Katarzyna Zachwieja, Ilona Zagozdzon, Jörg Dötsch, Franz Schaefer, Max Christoph Liebau, Alexandra Potemkina, Nadejda Ranguelov, Laure Collard, Aurélie De Mul, Markus Feldkoetter, Tomas Seeman, Jakub Zieg, Julia Thumfart, Franziska Grundmann, Björn Buchholz, Lars Pape, Oliver Gross, Ludwig Patzer, Raphael Schild, Dieter Haffner, Wanja Bernhardt, Elke Wuehl, Michael Henn, Jan Halbritter, Günter Klaus, Felix Lechner, Bärbel Lange-Sperandio, Barbara Uetz, Marcus Benz, Jens König, Hagen Staude, Donald Wurm, Martin Bald, Neveen A. Soliman, Gema Ariceta, Juan David Gonzalez Rodriguez, Francisco de la Cerda Ojeda, Jerome Harambat, Bruno Ranchin, Marc Fila, Claire Dossier, Olivia Boyer, Matko Marlais, Fotios Papachristou, Francesca Mencarelli, Antonio Mastrangelo, Luisa Murer, Francesco Emma, Dovile Ruzgiene, Katarzyna Taranta-Janusz, Irena Balasz-Chmielewska, Monika Miklaszewska, Malgorzata Stanczyk, Przemyslaw Sikora, Claudia Kowalewska, Maria Szczepanska, Ana Teixeira, Oliver Dunand, Andreea Rachisan, Dušan Paripović, Larisa Prikhodina, Houweyda Jilani, Aysun Karabay Bayazit, Alper Soylu, Cengiz Candan, Lale Sever, Sevinc Emre, Neslihan Cicek, Nurver Akinci, Sevgi Mir, Hakan M. Poyrazoğlu, Yilmaz Tabel, Hulya Nalcacioglu

**Affiliations:** 1 Department of Pediatrics, University Hospital Cologne and University of Cologne, Faculty of Medicine, Kerpener Str. 62, 50937 Cologne, Germany; 2 Institute of Medical Biometry and Informatics, University of Heidelberg, Heidelberg, Germany; 3 Department of Paediatrics and Adolescent Medicine, Medical University Vienna, Vienna, Austria; 4 Department of Pediatric Nephrology, Cukurova University Faculty of Medicine, Adana, Turkey; 5 Department of Pediatrics II, University Hospital Essen, Essen, Germany; 6 Pediatric Nephrology, Center for Paediatric and Adolescent Medicine, University Medical Clinic, Mainz, Germany; 7 Department of Pediatric Nephrology, Erciyes University, Faculty of Medicine, Kayseri, Turkey; 8 Division of Pediatric Nephrology, Department of Pediatrics, Hacettepe University Faculty of Medicine, Ankara, Turkey; 9 Division of Pediatric Nephrology, Department of Pediatrics, Dubai Hospital, Dubai, United Arab Emirates; 10 Department of Pediatrics and Adolescent Medicine, University of Erlangen-Nürnberg (FAU), Erlangen, Germany; 11 Department of General Pediatrics and Hematology/Oncology, Children’s University Hospital, Tübingen, Germany; 12 Division of Pediatric Nephrology, Research and Training Hospital, Marmara University, Istanbul, Turkey; 13 Department of Internal Medicine IV, Medical Center, University of Freiburg, Medical Faculty, Freiburg, Germany; 14 Department of Pediatric Nephrology, Ali-Asghar Children Hospital, Ali-Asghar Clinical Research Development Center (AACRDC), Iran University of Medical Sciences, Tehran, Iran; 15 Clinic of Children Diseases, Institute of Clinical Medicine, Vilnius University, Vilnius, Lithuania; 16 Pediatric Radiology, Institute of Diagnostic and Interventional Radiology, University Hospital of Cologne, Cologne, Germany; 17 Pediatric Nephrology, Dialysis and Transplant Unit, Department of Woman and Child Health, Azienda Ospedaliera-University of Padova, Padova, Italy; 18 Division of Nephrology, Department of Pediatric Subspecialties, Bambino Gesù Children’s Hospital, IRCCS, Rome, Italy; 19 PKD Research Group, Department of Development and Regeneration, KU Leuven, Leuven, Belgium; 20 Department of Pediatric Nephrology, University Hospitals Leuven, Leuven, Belgium; 21 Department of Nephrology, University Children’s Hospital, Belgrade, Serbia; 22 Pediatric Nephrology Department, Ondokuz Mayis University Faculty of Medicine, Samsun, Turkey; 23 Department of Nephrology, University Children’s Hospital, University Medical Centre Ljubljana, Ljubljana, Slovenia; 24 UCL Great Ormond Street Hospital for Children Institute of Child Health, UCL, London, UK; 25 First Department of Pediatrics, School of Medicine, Faculty of Health Sciences, Aristotle University of Thessaloniki, Hippokratio Hospital, Thessaloniki, Greece; 26 Clinic for Children and Adolescents, Hospital St. Georg, Leipzig, Germany; 27 Pediatric Nephrology Department, Istanbul Faculty of Medicine, Istanbul University, Istanbul, Turkey; 28 Department of Pediatric Nephrology and Hypertension, Faculty of Medicine, Jagiellonian University Medical College, Kraków, Poland; 29 Department of Nephrology and Hypertension of Children and Adolescents, Medical University of Gdansk, Gdańsk, Poland; 30 Division of Pediatric Nephrology, Center for Pediatrics and Adolescent Medicine, University of Heidelberg, Heidelberg, Germany; 31 Center for Molecular Medicine, University Hospital Cologne and University of Cologne, Faculty of Medicine, Cologne, Germany; 32 Department of Pediatrics, Université Catholique de Louvain Medical School, Saint-Luc Academic Hospital, Brussels, Belgium; 33 Centre de référence de Néphrologie Pédiatrique Sud, Clinique de l’Espérance, Montegnee, Belgium; 34 Unité Romande de Néphrologie Pédiatrique, Hôpitaux Universtaires de Genève, Geneva, Switzerland; 35 Pediatric Nephrology Unit, University Children’s Hospital, Zurich, Switzerland; 36 Department of Pediatrics, Dr. von Hauner Children’s Hospital, University Hospital, LMU, Munich, Germany; 37 Department of Pediatrics, University Hospital Motol, 2nd Faculty of Medicine, Charles University Prague, Prague, Czech Republic; 38 Department of Pediatric Nephrology, Charité–Universitätsmedizin Berlin, Corporate Member of Freie Universität Berlin, Humboldt-Universität zu Berlin, and Berlin Institute of Health, Berlin, Germany; 39 Department II of Internal Medicine, University Hospital of Cologne, Cologne, Germany; 40 Department of Nephrology and Hypertension, University of Erlangen-Nürnberg, Erlangen, Germany; 41 Clinic for Nephrology and Rheumatology, University Medical Center Goettingen, Göttingen, Germany; 42 Children’s Hospital St. Elisabeth and St. Barbara, Halle (Saale), Germany; 43 University Children’s Hospital, University Medical Center Hamburg Eppendorf, Hamburg, Germany; 44 Department of Pediatric Kidney, Liver and Metabolic Diseases, Hannover Medical School, Hannover, Germany; 45 Nephrology Clinic Hannover, Hannover, Germany; 46 Division of Nephrology, University Hospital Leipzig, Leipzig, Germany; 47 KfH Center of Paediatric Nephrology, University Hospital of Marburg, Marburg, Germany; 48 Pediatric Nephrology, Children’s Hospital, Memmingen, Germany; 49 KfH Center of Pediatric Nephrology, Children’s Hospital Munich Schwabing, Munich, Germany; 50 Klinik für Kinder-und Jugendmedizin, Klinikum Dritter Orden, Munich, Germany; 51 Department of General Pediatrics, University Hospital Muenster, Münster, Germany; 52 Pediatric Nephrology, University Children’s Hospital Rostock, Rostock, Germany; 53 Department of Pediatrics, Klinikum Saarbrücken, Saarbrücken, Germany; 54 Department of Pediatric Nephrology, Klinikum Stuttgart, Olga Children’s Hospital, Stuttgart, Germany; 55 Department of Pediatrics, Center of Pediatric Nephrology and Transplantation, Kasr Al Ainy School of Medicine, Cairo University, Cairo, Egypt; 56 Department of Pediatric Nephrology, University Hospital Vall d’Hebron, Barcelona, Spain; 57 Hospital General Universitario Murcia, Murcia, Spain; 58 Hospital Virgen del Rocío, Seville, Spain; 59 Department of Pediatrics, Bordeaux University Hospital, Bordeaux, France; 60 Pediatric Nephrology Unit, Hôpital Femme Mère Enfant, Hospices Civils de Lyon, Centre de référence maladies rénales rares, Bron, France; 61 Pediatric Nephrology Unit, CHU Arnaud de Villeneuve-Université de Montpellier, Montpellier, France; 62 Service de Néphrologie Pédiatrique, Hôpital Robertdebré, Paris, France; 63 Department of Pediatric Nephrology and Kidney Transplantation, Necker Hospital, Paris, France; 64 Nephrology and Dialysis Unit, Department of Pediatrics, Azienda Ospedaliero Universitaria Sant’Orsola-Malpighi, Bologna, Italy; 65 Pediatric Nephrology, Dialysis and Transplant Unit, Fondazione IRCCS Ca Granda, Ospedale Maggiore Policlinico, Milan, Italy; 66 Department of Paediatrics and Nephrology, Medical University of Bialystok, Białystok, Poland; 67 Department of Pediatrics, Immunology and Nephrology, Polish Mother’s Memorial Hospital Research Institute, Lodz, Poland; 68 Department of Pediatric Nephrology, Medical University of Lublin, Lublin, Poland; 69 The Children’s Memorial Health Institute, Warsaw, Poland; 70 Department of Pediatrics, Faculty of Medical Sciences in Zabrze, SUM in Katowice, Katowice, Poland; 71 Centro Materno-Infantil do Norte, Centro Hospitalar do Porto, Porto, Portugal; 72 Pediatric Nephrology Unit, CHU La Réunion site Felix GUYON, Saint-Denis, Réunion; 73 Department of Pediatrics, “Iuliu Haţieganu” University of Medicine and Pharmacy, Cluj-Napoca, Romania; 74 Department of Inherited and Acquired Kidney Diseases, Research Clinical Institute for Pediatrics n.a. acad. Y. E. Veltishev, Pirogov Russian National Research Medical University, Moscow, Russia; 75 Service des Maladies Congénitales et Héréditaires, CHU Mongi Slim La Marsa, Sidi Daoud La Marsa, Tunis, Tunisia; 76 Department of Pediatric Nephrology, Dokuz Eylul University Medical Faculty, Balcova, Izmir, Turkey; 77 Division of Pediatric Nephrology, Göztepe Hospital, Istanbul Medeniyet University, Istanbul, Turkey; 78 Department of Pediatric Nephrology, Cerrahpaşa School of Medicine, Istanbul University, Istanbul, Turkey; 79 Division of Pediatric Nephrology, Sisli Etfal Training and Research Hospital, Istanbul, Turkey; 80 Department of Pediatric Nephrology, Ege University Medical Faculty, Izmir, Turkey; 81 Department of Pediatric Nephrology, Faculty of Medicine, İnönü University, Malatya, Turkey; 82 Division of Pediatric Nephrology, Faculty of Medicine, Ondokuz Mayis University, Samsun, Turkey

**Keywords:** Chronic kidney disease, Paediatric kidney disease, Polycystic kidney disease

## Abstract

Autosomal recessive polycystic kidney disease (ARPKD) is characterized by bilateral fibrocystic changes resulting in pronounced kidney enlargement. Impairment of kidney function is highly variable and widely available prognostic markers are urgently needed as a base for clinical decision-making and future clinical trials. In this observational study we analyzed the longitudinal development of sonographic kidney measurements in a cohort of 456 ARPKD patients from the international registry study ARegPKD. We furthermore evaluated correlations of sonomorphometric findings and functional kidney disease with the aim to describe the natural disease course and to identify potential prognostic markers. Kidney pole-to-pole (PTP) length and estimated total kidney volume (eTKV) increase with growth throughout childhood and adolescence despite individual variability. Height-adjusted PTP length decreases over time, but such a trend cannot be seen for height-adjusted eTKV (haeTKV) where we even observed a slight mean linear increase of 4.5 ml/m per year during childhood and adolescence for the overall cohort. Patients with two null *PKHD1* variants had larger first documented haeTKV values than children with missense variants (median (IQR) haeTKV 793 (450–1098) ml/m in Null/null, 403 (260–538) ml/m in Null/mis, 230 (169–357) ml/m in Mis/mis). In the overall cohort, estimated glomerular filtration rate decreases with increasing haeTKV (median (IQR) haeTKV 210 (150–267) ml/m in CKD stage 1, 472 (266–880) ml/m in stage 5 without kidney replacement therapy). Strikingly, there is a clear correlation between haeTKV in the first eighteen months of life and kidney survival in childhood and adolescence with ten-year kidney survival rates ranging from 20% in patients of the highest to 94% in the lowest quartile. Early childhood haeTKV may become an easily obtainable prognostic marker of kidney disease in ARPKD, e.g. for the identification of patients for clinical studies.

## Introduction

Autosomal recessive polycystic kidney disease (ARPKD) is a severe hepatorenal disorder that typically becomes symptomatic early in life or even prenatally. The classic phenotype involves kidney enlargement due to the development of ubiquitous renal microcysts^1^. There is pronounced clinical variability both for the renal and the hepatic clinical phenotype that can only partially be explained by underlying genetic variants in the main affected gene *PKHD1*^1–3^. Treatment in ARPKD currently remains symptomatic^2,4^. This is partly due to the fact that primary endpoints for interventional clinical trials have not yet been established and that risk cohorts have not been defined. For autosomal dominant polycystic kidney disease (ADPKD) total kidney volume (TKV) and height-adjusted total kidney volume (haTKV) serve as surrogate risk markers of disease progression in adults^5^. For ARPKD a loose inverse correlation between TKV and kidney function has been observed in children and it is widely accepted that kidney growth patterns show differences between ARPKD and ADPKD^6–8^. In a sonographic study from 1995 following nine patients with the histological diagnosis of ARPKD, a decrease of kidney size with time was observed in five of the nine subjects^9^. Another study showed stable renal size in 16 ARPKD children surviving the neonatal period^8^. More recently, in two cohorts of 31 and 50 ARPKD patients, respectively, no clear correlation between eGFR slope and kidney size were detected^10,11^. Kidney volumes and lengths remained stable over time in one of the cohorts^11^. Overall, however, the natural history of kidney size in ARPKD and its correlation to kidney function remain incompletely understood.

Over the past years we have established an international ARPKD cohort study^12,13^. Using this observational longitudinal data we have previously identified antenatal sonographic markers as risk markers for early dialysis dependency in ARPKD^14^. In the current study we aimed to (1) characterize in detail the longitudinal courses of different kidney size measures during childhood and adolescence and to (2) evaluate a potential association of early height-adjusted estimated total kidney volume (haeTKV) with kidney survival in ARPKD on the basis of a well-characterized cohort of up to 456 patients. This improved understanding of clinical courses may serve as a base to identify ARPKD patients that are at risk of developing early kidney failure.

## Results

### Patients

At the timepoint of data extraction 543 patients were included in ARegPKD. 456 patients had at least one ultrasound prior to a first nephrectomy. The patients´ characteristics are displayed in Table 1.

**Table 1:** Patient characteristics.

	Total (n = 456)
**Clinical information**	
Sex, n (%)	
Male	230/456 (50%)
Female	226/456 (50%)
Last available CKD stage, n (%)	
G1	102/446 (23%)
G2	95/446 (21%)
G3	92/446 (21%)
G4	37/446 (8%)
G5 without KRT	19/446 (4%)
G5 with KRT	101/446 (23%)
Age at first visit (yrs), n	456
Median (IQR)	1.7 (0.2–7.4)
Age at last visit (yrs), n	456
Median (IQR)	8.2 (3.6–14.6)
Number of visits, n	456
Median (IQR)	3.0 (2.0–7.0)
Follow-up time, n	456
Median (IQR)	2.3 (0.4–7.3)
Age at initial diagnosis (yrs), n	418
Median (IQR)	0.3 (0.1–1.6)
Gestational age at birth (weeks), n	341
Median (IQR)	38.0 (36.0–39.0)
Perinatal assisted breathing, n (%)	94/402 (23%)
Patients with documented nephrectomies, n (%)	45/456 (10%)
Age at first or bilateral nephrectomy (yrs), n	45
Median (IQR)	1.1 (0.1–5.6)
**Genetic information**	
Genetic confirmation, n (%)	
No *PKHD1* testing, no *PKHD1* variant detected	260/456 (57%)
ARPKD genetically confirmed	116/456 (25%)
ARPKD genetically probable	35/456 (8%)
ARPKD genetically unknown	45/456 (10%)
Functional *PKHD1* variant classifications, n (%)	
Null/null	11/196 (6%)
Null/missense	50/196 (26%)
Missense/missense	85/196 (43%)
Others	24/196 (12%)
Single variant	26/196 (13%)

Percentages have been rounded to whole numbers. Months (mo), years (yrs).

### Development of pole-to-pole length and eTKV in ARPKD over time and normalization for body height

**Figure 1:**
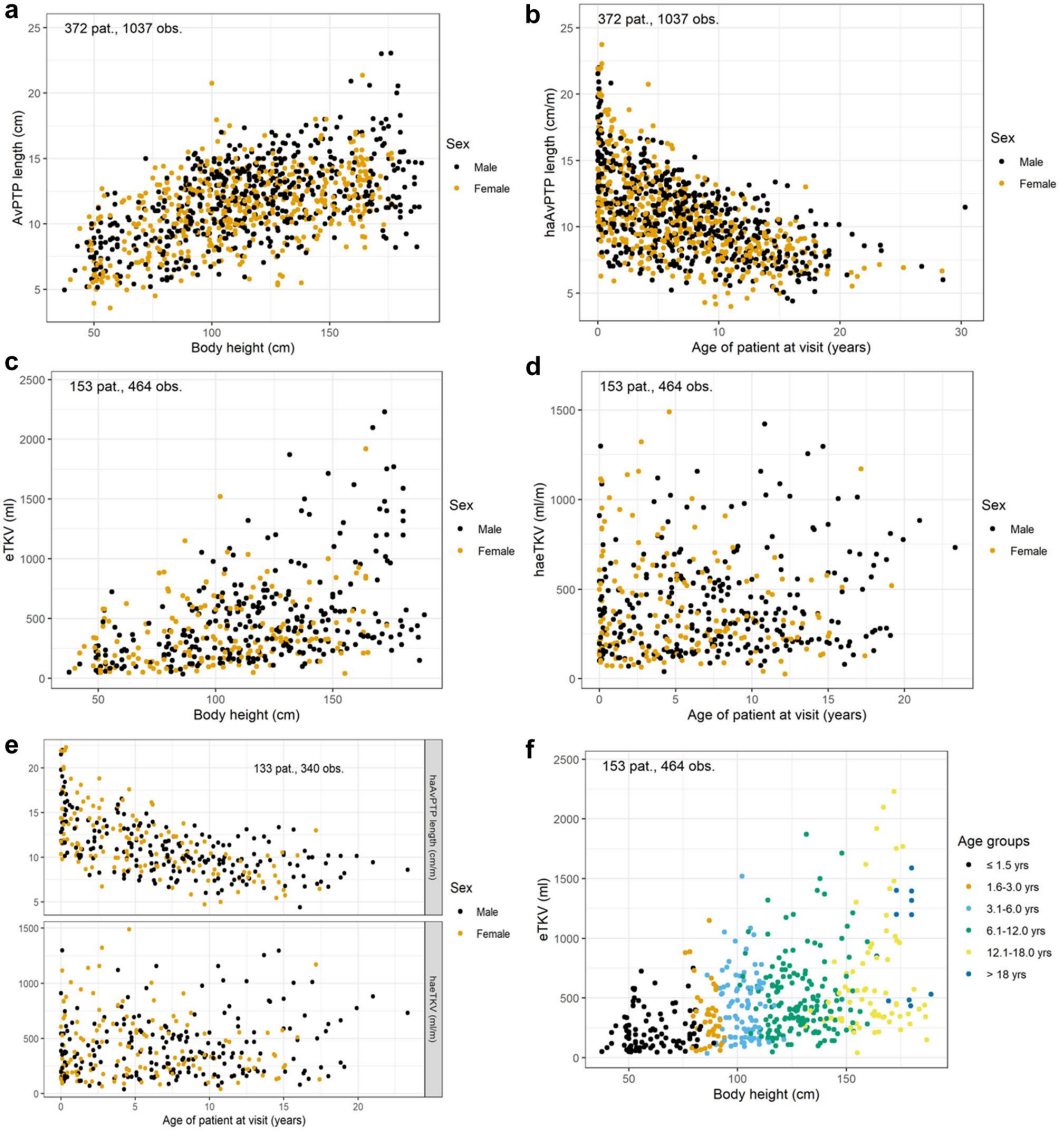
Sonographic findings over time: Average kidney pole-to-pole length (avPTP length) increases with body height (**a**), height-adjusted avPTP length decreases with age (**b**). Estimated total kidney volumes (eTKV) increase with body height (**c**), height-adjusted value (haeTKV) distribution is stable, although variable, in children (**d**). These findings were confirmed in a subset of patients in whom both haPTP length and haeTKV were available (**e**). The distribution of eTKV showed a similar relation to body height in different age groups (**f**).

The longitudinal description of sonographic pole-to-pole length (PTP length) is based on up to 1205 datapoints from 408 patients. The lengths of the right and the left kidney were highly correlated (Supplementary Fig. S1 online). The average absolute PTP length (AvPTP length) increased with body length (Fig. 1a) whereas height-adjusted AvPTP length (haAvPTP length) decreased with increasing age (Fig. 1b).

To evaluate the validity of sonographic PTP length measurements, comparisons with MRI or CT assessments were performed in a small subset of patients in which both analyses were available within a short time frame (Supplementary Fig. S1 online). Overall, there was a good correlation with slight overestimation of PTP length in very large kidneys (PTP length > 15 cm) by ultrasound.

The longitudinal description of sonographic estimated kidney volume (eTKV) is based on up to 508 datapoints from 161 patients using the ellipsoid formula. Again, a close correlation between the right and the left kidney was noted (Supplementary Fig. S1 online). While eTKV increased with height (Fig. 1c), height-adjusted eTKV (haeTKV) was less dependent on age throughout childhood and adolescence and remained rather stable with a slight mean linear increase of 4.5 ml/m per year during childhood and adolescence for the overall cohort (Fig. 1d). This difference between haeTKV and haAvPTP length was confirmed in a subset of 133 patients with 340 datapoints in whom both haAvPTP lengths and haeTKV values were available (Fig. 1e).

The two transversal kidney diameters increased slightly with age in absolute terms but showed decrease with age when adjusted for height (Supplementary Fig. S2 online).

To investigate why we saw a decrease of haAvPTP lengths but not of haeTKV over time we changed the height-adjustment. To account for the different dimensionality between volume and length we divided by $\sqrt[3]{body\, height}$—resulting in an alternative height-adjustment of AvPTP length (“alternative haAvPTP”). This revealed more constant values over time (Supplementary Fig. S2 online). Consistently, eTKV shows a greater than linear increase with increasing AvPTP lengths (Supplementary Fig. S3 online). In more than 97% of all documented height-adjusted measurements prior to nephrectomy, measurements of both kidneys were available.

### eTKV correlation to body height in different age groups and to genetic classes

We next stratified the eTKV findings according to age groups and observed comparable eTKV patterns for the different age groups (Fig. 1f). Separation of eTKV according to age and height showed a comparable distribution of subgroups as separation according to age and weight (Supplementary Fig. S3 online).

**Figure 2:**
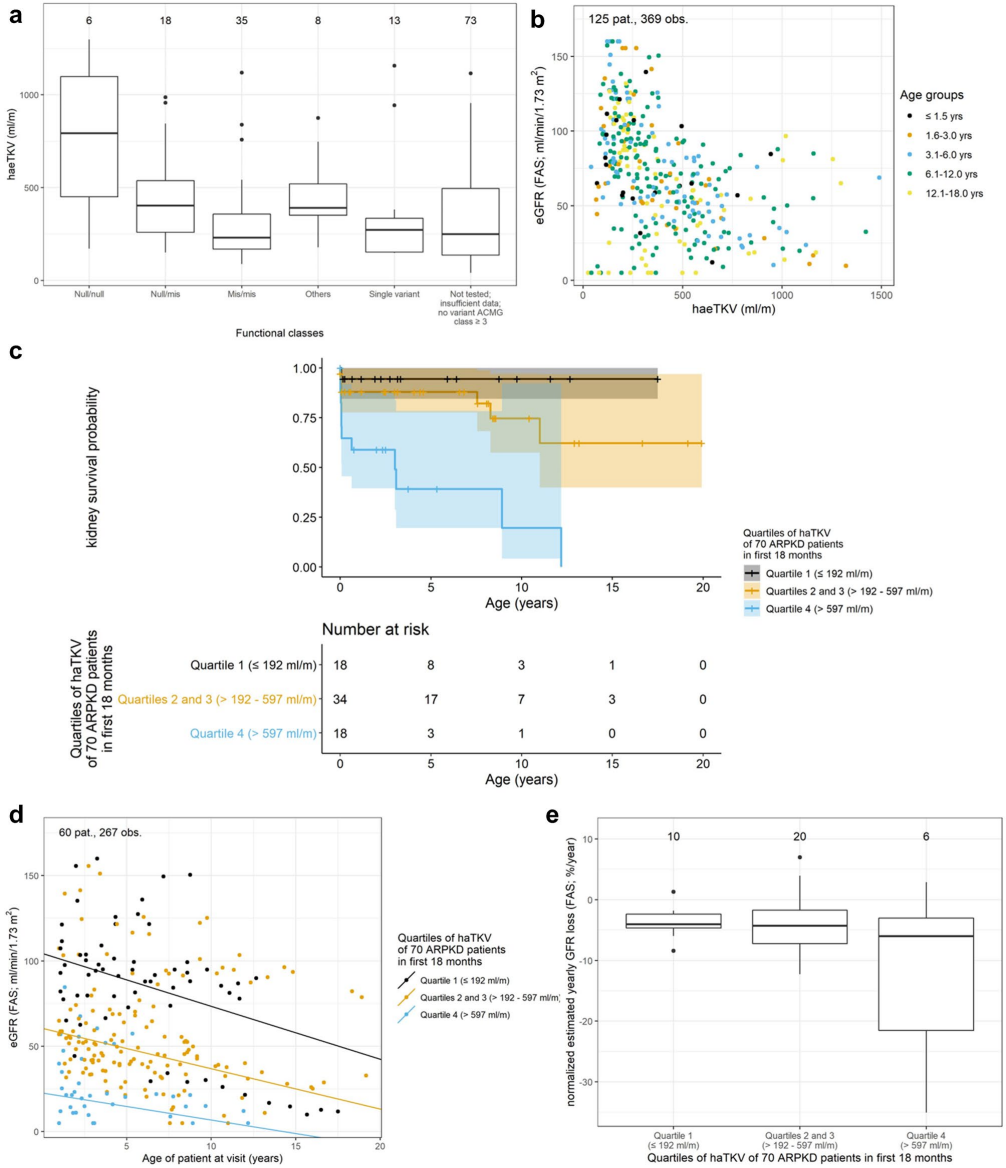
Correlation of haeTKV to genetics and kidney function: HaeTKV values for different groups of genotypes. The first documented haeTKV value was used for each patient (**a**). eGFR and haeTKV show a loose inverse relation in the total cohort (**b**). Kaplan–Meier of kidney survival. The highest quartile of haeTKVmax18values shows poorest kidney outcome (**c**). Scatter plot and regression lines of eGFR values of haeTKVmax18 patients stratified according to haeTKVmax18 quartiles (**d**). Normalized yearly eGFR loss of haeTKVmax18 patients stratified according to haeTKVmax18 quartiles. Only patients with at least 3 visits spanning at least one year were used to obtain valid estimations (**e**).

Comparing haeTKV values according to the level of genetic certainty of the diagnosis did neither reveal a clear age-related pattern, nor in the absolute values (Supplementary Fig. S4 online). However, patients with two null *PKHD1* variants had larger first documented haeTKV values than children with one or two missense variants (median (IQR) haeTKV 793 (450–1098) ml/m in Null/null, 403 (260–538) ml/m in Null/mis, 230 (169–357) ml/m in Mis/mis; Fig. 2a). There was no clear clustering of haeTKV values with regards to *PKHD1* functional classes along the age spectrum (Supplementary Fig. S4 online). For the alternative haAvPTP (cm/$\sqrt[3]{m}$), we did not observe a correlation with the genetic classification (Supplementary Fig. S5 online).

### haeTKV correlation to kidney function

An inverse correlation between haeTKV and eGFR was found (Fig. 2b), resulting in an increase of median haeTKV values with CKD stage during childhood and adolescence without an apparent correlation to age (median (IQR) haeTKV 210 (150–267) ml/m in stage 1, 258 (158–437) ml/m in stage 2, 424 (255–530) ml/m in stage 3, 632 (331–878) in stage 4 and 472 (266–880) ml/m in stage 5 without KRT; Supplementary Fig. S6 online).

To evaluate whether haeTKV at an early timepoint may help to predict the risk for early loss of kidney function, patients were stratified according to their highest documented haeTKV value within the first 18 months of life (haeTKVmax18). Kidney survival according to haeTKVmax18 quartile was analyzed in 70 prospectively followed children. The characteristics of this subcohort are summarized in Supplementary Table S1 online. More patients in the upper haeTKVmax18 quartile (> 597 ml/m) showed progression to KRT than patients in the two middle quartiles (> 192–597 ml/min; p < 0.001) or the lower quartile (≤ 192 ml/min; p < 0.001). 10-year-KRT-free survival was 20% in the upper quartile, 75% in the two middle quartiles, and 94% in the lower haeTKVmax18 quartile, respectively (Fig. 2c). For haAvPTP length and alternative haAvPTP length the correlation to kidney function was less clear (p = 0.1 for both haAvPTP length and alternative haAvPTP length upper vs. lower quartile) than for haeTKV (Supplementary Fig. S7 online).

Mean eGFR at the end of the first year of life was 21, 58 and 101 ml/min/1.73 m^2^ in the highest, the two middle and the lowest haeTKVmax18 quartile, respectively (Fig. 2d). Normalized estimated yearly eGFR loss among the subgroups showed no major differences (Fig. 2e). Analysis of longitudinal follow-up data suggested a loose inverse correlation between longitudinal haeTKV changes and changes in normalized eGFR in the haeTKVmax18 cohort (Supplementary Fig. S8 online).

## Discussion

We present longitudinal descriptive imaging data on the development of kidney length and kidney volume during the course of ARPKD and identify maximal haeTKV within the first 18 months of life as a potential prognostic marker for kidney survival in ARPKD. This study represents the largest collection of renal imaging data in ARPKD, with more than 1000 datapoints for specific questions. Our cohort covers the entire pediatric age range.

The analysis of our dataset substantially expands the existing knowledge both on the natural history of kidney growth in ARPKD as well as on the correlations between kidney size and kidney function. We observed significant growth of the polycystic kidneys in all dimensions and total volume with time. Yet, while height-adjusted lengths decrease in all dimensions, haeTKV remained rather constant. For healthy children it has been reported that haAvPTP length also slightly decreases over time but this effect is by far less pronounced than in ARPKD. HaeTKV remains relatively stable in healthy children^15,16^. Thus, ARPKD follows general growth patterns. It will be of interest to compare the ARPKD kidney growth patterns observed here with other etiologies of renal dysplasia in future studies. Severe variants in the *PKHD1* gene seem to be associated with highest haeTKV values in ARPKD.

Our findings have multiple important implications. Firstly, the data do not support a concept that kidney size or kidney volume would remain unchanged in ARPKD or would even become smaller during the course of the disease in childhood and adolescence. Indeed, we found that absolute kidney size increases over time although the growth did not show the dynamics seen in ADPKD and the relative increase of kidney PTP length compared to body height was smaller. Our data thus fit to previous reports^8,11^. With respect to a preference of kidney length or kidney volume, haeTKV may be a better marker to compare kidney size in ARPKD children than haPTP length throughout childhood and adolescence. Our numbers in adult patients remain too small to draw reliable conclusions on the courses of radiological findings or potential correlations to kidney function. In the current study we have therefore focussed on pediatric patients. We have previously described the clinical findings of our cohort of young adults^17^. Interestingly, in the very small number of adult patients we had found smaller native kidneys, which is in accordance with the clinical experience of many centers. From the available data we cannot yet fully explain this apparent discrepancy to the data presented in this manuscript describing a stable relationship between eTKV and height over time during childhood and adolescence. It seems plausible that a selection bias towards less severely affected patients that survived into adulthood with their native kidneys in place (i.e. without the necessity of bilateral nephrectomies of native ARPKD kidneys) may have contributed to the perception of smaller kidneys in adult patients with ARPKD. Larger studies with follow-up on adolescents and adult patients of severely affected surviving ARPKD children not undergoing nephrectomy will be required to close this gap of knowledge.

Secondly, the data support the value of ultrasound-based measurement of renal size in ARPKD as a standard for most of the patients and also as a standard for clinical studies. Other than in ADPKD, where kidney volumes in mostly adult patients are assessed by computed tomography or magnetic resonance imaging^5^, ultrasound is the method of choice for kidney examination in pediatric patients due to its wide availability, excellent risk–benefit balance and its applicability without sedation of the infant or child^7^. Our data showed very good right-left correlation for PTP lengths with good correlation for kidney volumes. Exact sonographic measurement of very large kidneys may be a challenge, but comparison with MRI measurements suggested that ultrasound is sufficiently accurate for clinical and research purposes in children with ARPKD. While some centers seem to have a preference in documentation of kidney size by either rather PTP lengths or rather kidney volumes, our analysis addresses both approaches and allows to compare them.

Thirdly, we establish a novel link between early haeTKV and kidney survival in ARPKD that may become a helpful prognostic marker for counselling families and identifying patients at special risk of poor kidney survival e.g. for clinical trials. We confirmed a previously-reported loose inverse correlation between haeTKV and eGFR in our larger cohort^6,11^. Our longitudinal data, however, also allowed us to follow kidney disease progression in specific patient subgroups. Higher haeTKV values in the first 18 months of life were associated with poorer kidney survival already in childhood and adolescence. Importantly, haeTKV showed better prognostic discrimination than either haAvPTP length or alt.haAvPTP. Three-dimensional assessment thus adds relevant information to unidimensional description of kidney length and should be used as clinical standard. This association of early haeTKV with kidney survival needs to be validated in an independent ARPKD patient cohort.

Finally, the data suggest that the patients with the largest kidneys do not even reach the same eGFR values at the end of the first year of life observed in children with less severe phenotypes. Early structural changes may be decisive for poor kidney survival in severely affected ARPKD patients. If this concept holds true, therapeutic pharmacological intervention would have to start very early in life for major clinical impact. Depiction of changes of haeTKV vs. eGFR over time may be suggestive of a loose inverse relationship, but more data is required for confirmation. Our data may point to a prognostic and potentially predictive value of haeTKV for ARPKD early in life but more longitudinal observations in independent cohorts and verification of this potential in multivariable analyses after implementation of all qualifying risk markers will be needed. Especially, other risk factors respectively confounders like genetics, prematurity, postnatal ventilation, eGFR at initial presentation, arterial hypertension or conduction of unilateral nephrectomy need to be implemented in more detailed follow-up work. A comprehensive analysis of all of these aspects was beyond the scope of this initial description of kidney size courses in ARPKD. Importantly, the eGFR slopes observed in our patients are in keeping with previous reports from two pediatric ARPKD cohorts^11,18^.

Several limitations of our study deserve to be mentioned. The structure of the ARegPKD registry may be associated with an inclusion bias as patients with the most severe early phenotypes and e.g. perinatal demise may not be included. Furthermore, patients with late or atypical courses may be missed. Our study focusses on the pediatric age resulting in small numbers of patients with this rare disease at the end of follow-up in survival analyses. Data inclusion into ARegPKD occurs on a voluntary basis with the clinical diagnosis of ARPKD, resulting in incomplete available data for some patients, including genetic disease confirmation. We chose to present the existing data as collected rather than imputing missing data. Furthermore, sonographic measurements were conducted by different investigators, central reading of sonographic information was not possible in this setting of investigating a rare disease. Estimated TKV was calculated in some patients based on lengths in three dimensions but was directly entered into the database in other patients. Even with the mentioned limitation the dataset with its substantial numbers offers novel and reliable insights.

In summary, we describe the longitudinal course of kidney length and estimated kidney volume in a very large group of pediatric ARPKD patients. Our findings can serve as a starting point to further evaluate haeTKV in the first 18 months of life as a clinical prognostic marker in ARPKD both for clinical practice as well as for clinical studies on ARPKD.

## Methods

The international observational cohort study ARegPKD follows patients with the clinical diagnosis of ARPKD according to a previously published protocol^12^. In brief, real life clinical data covering different aspects of ARPKD are collected pro- and retrospectively with automated data entry checks and regular quality control. This includes a detailed set of radiological data. Informed consent was obtained from all subjects or, if subjects are under 18, from a parent and/or legal guardian according to applicable local regulations. The study protocol was approved by the Ethics Committee of the Faculty of Medicine of Cologne University and the Institutional Review Boards of participating sites. ARegPKD is in accordance with the ethical standards of the institutional and/or national research committee and with the 1964 Helsinki declaration and its later amendments or comparable ethical standards. Data analysis was performed on the ARegPKD dataset available on March 2019, with genetic data available as of April 2019.

Estimation of glomerular filtration rate (eGFR) was restricted to patient visits from 1 to 18 years and was based on the full age spectrum formula^19^. Categorization into CKD stages was applied according to KDIGO classification. On kidney replacement therapy (KRT, on dialysis or after kidney transplantation (KTx)) eGFR values were set to 5 ml/min/1.73 m^2^ as indicated in detail below. An upper eGFR limit of 160 ml/min/1.73 m^2^ was defined and higher eGFR values were set to 160 ml/min/1.73 m^2^.

Data on pole-to-pole length of the kidneys (PTP length), transversal lengths in two dimensions, and estimated total kidney volume (eTKV) were collected. Kidney volume was estimated based on a simple ellipsoid formula (length x width x depth x π/6) in patients with available measurements. eTKV represents the sum of left and right estimated kidney volume (eKV). Average PTP length (AvPTP length) was defined as the sum of both PTP lengths divided by two. If only one eKV or PTP length value was available, this value was also chosen for the other side in patients prior to nephrectomy. For comparison of ultrasound and magnetic resonance (MR) or computer tomography (CT) imaging, paired examinations obtained within three months in the first year of life and within six months beyond the first year of life were included. Height-adjustment for eTKV (haeTKV, defined as eTKV/body height in m) or AvPTP length (haAvPTP length, defined as AvPTP length/body height in m) was applied for patients with date of height and ultrasound measurement within three months for patients younger than three years or within 10% of the current age for older patients. Alternative haAvPTP length was defined as AvPTP/$\sqrt[3]{\mathrm{body\, height\, in\, m}}$. For categorization into haeTKV quartiles the highest documented values for haeTKV in the first 18 months of life (haeTKVmax18) was used. Radiological findings were stratified according to the age groups ≤ 1.5 years of age, > 1.5–3 years, > 3–6 years, > 6–12 years and > 12–18 years.

Correlations of TKV and eGFR were only calculated on cases without nephrectomies. All eGFR values were used in the analysis of the haeTKVmax18 cohort since nephrectomy-related reduction in nephron mass was considered a direct consequence of large size. Data on follow-up time indicates follow-up for evaluation of kidney survival and eGFR of patient subgroups in haeTKVmax18 quartiles.

For genetic analyses all reported *PKHD1* variants were classified according to the revised criteria of the American College of Medical Genetics (ACMG)^20^. The genotypes were assigned to functional classes termed null variants (nonsense and frameshift variants, canonical splice-site variants, whole gene deletions) or missense variants (≥ ACMG class 3). Patients with only a single variant ≥ ACMG class 3 and those with any other combination were grouped independently. According to molecular genetic diagnostic certainty, patients were sub-grouped in the classes “Confirmed” (≥ 2 *PKHD1* variants detected, with at least two ≥ ACMG class 4), “Probable” (≥ 2 *PKHD1* variants, only one ≥ ACMG class 4), and “Unknown” (≥ 2 *PKHD1* ACMG class 3 or only one *PKHD1* variant ≥ ACMG class 3). All other patients (*PKHD1* variants ACMG 1 or 2, no documented *PKHD1* variants in case of *PKHD1* sequencing, no *PKHD1* sequencing) were grouped together.

### Statistics

All statistical analyses were performed using R, version 4.0.1^21^. Continuous variables were described using the number of non-missing values, mean and standard deviation (SD) as well as median and interquartile range (IQR). For binary or categorical variables, absolute and relative frequencies were provided. Kidney survival was estimated by the Kaplan–Meier method. Estimated GFRs at the end of the first year of life within haeTKVmax18 quartiles were estimated by a linear regression using eGFR as dependent variable and age at eGFR measurement (continuous), haeTKVmax18 group (categorical) and the interaction of age and haeTKVmax18 group as independent variables. The patient ID was included as random factor with an interaction with age, thus considering the variability between patients and assuming individual eGFR-age relationships for each patient. For this calculation eGFR was defined as 5 ml/min/1.73 m^2^ for children on KRT. For longitudinal descriptions of children on KRT only the first visits after the end of the first year of life were included. Mean annual increase of haeTKV was estimated by a linear regression of haeTKV versus age including patient ID as random factor. Annual eGFR loss was estimated for each patient as the slope coefficient of a linear regression of eGFR versus age. For this, only patients with at least three visits spanning at least one year were used to obtain valid estimations. Annual eGFR loss was normalized as percental yearly change. Data completeness varied by variable and timepoint of data collection resulting in different numbers of informative cases for every subanalysis. The specific numbers of informative patients and observations are indicated in each figure panel. Missing data were handled by pairwise deletion of cases that had missing values necessary for the respective analysis. No imputation of missing values was performed.

## Supplementary Information

Supplementary Information.

## Data Availability

The datasets generated during and/or analysed during the current study are available from the corresponding author on reasonable request.
