# Supplementary material for: Early childhood height-adjusted total kidney volume as a risk marker of kidney survival in ARPKD
Source: Sci Rep. 2021 Nov 4;11:21677. doi: 10.1038/s41598-021-00523-z (PMC8568977; doi:10.1038/s41598-021-00523-z)
Supplement: Supplementary file 1 — Supplementary Information. [file 41598_2021_523_MOESM1_ESM.pdf]

Early childhood height-adjusted total kidney volume as a risk marker of kidney survival in ARPKD

Kathrin Burgmaier MD[1], Samuel Kilian[2], Klaus Arbeiter MD[3], Bahriye Atmis MD[4], Anja Büscher MD[5], Ute Derichs MD[6], Ismail Dursun MD[7], Ali Duzova MD[8], Loai Akram Eid MD[9], Matthias Galiano MD[10], Michaela Gessner MD[11], Ibrahim Gokce MD[12], Karsten Haeffner MD[13], Nakysa Hooman MD[14], Augustina Jankauskiene MD[15], Friederike Körber MD[16], Germana Longo MD[17], Laura Massella MD[18], Djalila Mekahli MD,PhD[19,20], Gordana Miloševski-Lomić MD[21], Hulya Nalcacioglu MD[22], Rina Rus MD[23], Rukshana Shroff MD[24], Stella Stabouli MD[25], Lutz T. Weber MD[1], Simone Wygoda MD[26], Alev Yilmaz MD[27], Katarzyna Zachwieja MD[28], Ilona Zagozdzon MD, PhD[29], Jörg Dötsch MD[1], Franz Schaefer MD[30], Max Christoph Liebau MD[1,31]* & The ARegPKD consortium

Affiliations:
[1] Department of Pediatrics, University Hospital Cologne and University of Cologne, Faculty of Medicine, Cologne, Germany
[2] Institute of Medical Biometry and Informatics, University of Heidelberg, Heidelberg, Germany
[3] Department of Paediatrics and Adolescent Medicine, Medical University Vienna, Vienna, Austria
[4] Department of Pediatric Nephrology, Cukurova University Faculty of Medicine, Adana, Turkey
[5] Department of Pediatrics II, University Hospital Essen, Essen, Germany
[6] Pediatric Nephrology, Center for Paediatric and Adolescent Medicine, University Medical Clinic, Mainz, Germany
[7] Department of Pediatric Nephrology, Erciyes University, Faculty of Medicine, Kayseri, Turkey
[8] Department of Pediatrics, Division of Pediatric Nephrology, Hacettepe University Faculty of Medicine, Ankara, Turkey
[9] Department of Pediatrics, Division of Pediatric Nephrology, Dubai Hospital, Dubai, United Arab Emirates
[10] Department of Pediatrics and Adolescent Medicine, University of Erlangen-Nürnberg (FAU), Erlangen, Germany
[11] Department of General Pediatrics and Hematology/Oncology, Children's University Hospital Tuebingen, Germany
[12] Research and Training Hospital, Division of Pediatric Nephrology, Marmara University, Istanbul, Turkey
[13] Department of Internal Medicine IV, Medical Center, University of Freiburg, Medical Faculty, Freiburg, Germany
[14] Department of Pediatric Nephrology, Ali-Asghar Children Hospital, Ali-Asghar Clinical Research Development Center (AACRDC), Iran University of Medical Sciences, Tehran, Iran
[15] Clinic of Children Diseases, Institute of Clinical Medicine, Vilnius University, Vilnius, Lithuania
[16] Pediatric Radiology, Institute of Diagnostic and Interventional Radiology, University Hospital of Cologne, Cologne, Germany
[17] Pediatric Nephrology, Dialysis and Transplant Unit, Department of Woman and Child Health, Azienda Ospedaliera-University of Padova, Padova, Italy
[18] Division of Nephrology, Department of Pediatric Subspecialties, Bambino Gesù Children's Hospital, IRCCS, Rome, Italy
[19] Department of Development and Regeneration, PKD Research Group, KU Leuven, Leuven, Belgium

[20] Department of Pediatric Nephrology, University Hospitals Leuven, Leuven, Belgium
[21] Department of Nephrology, University children's Hospital, Belgrade, Serbia
[22] Ondokuz Mayis University Faculty of Medicine, Pediatric Nephrology Department, Samsun, Turkey
[23] University Medical Centre Ljubljana, University Children's Hospital, Dept of Nephrology, Ljubljana, Slovenia
[24] UCL Great Ormond Street Hospital for Children Institute of Child Health, UCL, London, UK
[25] First Department of Pediatrics, School of Medicine, Faculty of Health Sciences, Aristotle University of Thessaloniki, Hippokratio Hospital, Thessaloniki, Greece
[26] Clinic for Children and Adolescents, Hospital St. Georg, Leipzig, Germany
[27] Pediatric Nephrology Department, Istanbul Faculty of Medicine, Istanbul University, Istanbul, Turkey
[28] Department of Pediatric Nephrology and Hypertension, Faculty of Medicine, Jagiellonian University Medical College, Krakow, Poland
[29] Department of Nephrology and Hypertension of Children and Adolescents, Medical University of Gdansk, Gdansk, Poland
[30] Division of Pediatric Nephrology, Center for Pediatrics and Adolescent Medicine, University of Heidelberg, Heidelberg, Germany
[31] Center for Molecular Medicine, University Hospital Cologne and University of Cologne, Faculty of Medicine, Cologne, Germany

Running head:

Total kidney volume in ARPKD

***Corresponding author:**

Max Christoph Liebau, MD
Department of Pediatrics and Center for Molecular Medicine, University Hospital of Cologne
Kerpener Str. 62
50937 Cologne
Germany
e-mail: max.liebau@uk-koeln.de
Phone: +49-221-478-4359

Supplementary Table S1:

Clinical information	Total (n=70)
Sex, n (%)<br>- male<br>- female	 37/70 (53%)<br>33/70 (47%)
Last available CKD stage, n (%)<br>- G1<br>- G2<br>- G3<br>- G4<br>- G5 without KRT<br>- G5 with KRT	 11/69 (16%)<br>11/69 (16%)<br>19/69 (28%)<br>5/69 (7%)<br>6/69 (9%)<br>17/69 (25%)
Age at first visit (yrs), n<br>- median (IQR)	70<br>0.2 (0.1-0.5)
Age at last visit (yrs), n<br>- median (IQR)	70<br>5.0 (1.9-9.5)
Number of visits, n<br>- median (IQR)	70<br>5.5 (2.0-9.0)
Follow-up time, n<br>- median (IQR)	70<br>4.9 (1.5-9.0)
Age at initial diagnosis (yrs), n<br>- median (IQR)	68<br>0.1 (0.0-0.2)
Gestational age at birth (weeks), n<br>- median (IQR)	60<br>36.5 (34.0-38.0)
Perinatal assisted breathing, n (%)	38/63 (60%)
Patients with documented nephrectomies, n (%)	13/70 (19%)
Age at first or bilateral nephrectomy (yrs), n<br>- median (IQR)	13<br>0.1 (0.1-3.5)
Maximal haeTKV in first 18 months (ml), n<br>- median (IQR)	70<br>350.6 (192.1-597.0)
Genetic information	
Genetic confirmation, n (%)<br>- No *PKHD1* testing, no *PKHD1* variant detected<br>- ARPKD genetically confirmed<br>- ARPKD genetically probable<br>- ARPKD genetically unknown	 35/70 (50%)<br>22/70 (31%)<br>5/70 (7%)<br>8/70 (11%)
Functional *PKHD1* variant classifications, n (%)<br>- Null/null<br>- Null/missense<br>- Missense/missense<br>- Others<br>- Single variant	 6/35 (17%)<br>7/35 (20%)<br>13/35 (37%)<br>5/35 (14%)<br>4/35 (11%)

Supplementary Table S1: Patient characteristics of the analyzed cohort classified for maximal height-adjusted estimated total kidney volume in the first 18 months of life. Percentages have been rounded to whole numbers. Months (mo), years (yrs).

Supplementary Figure S1:

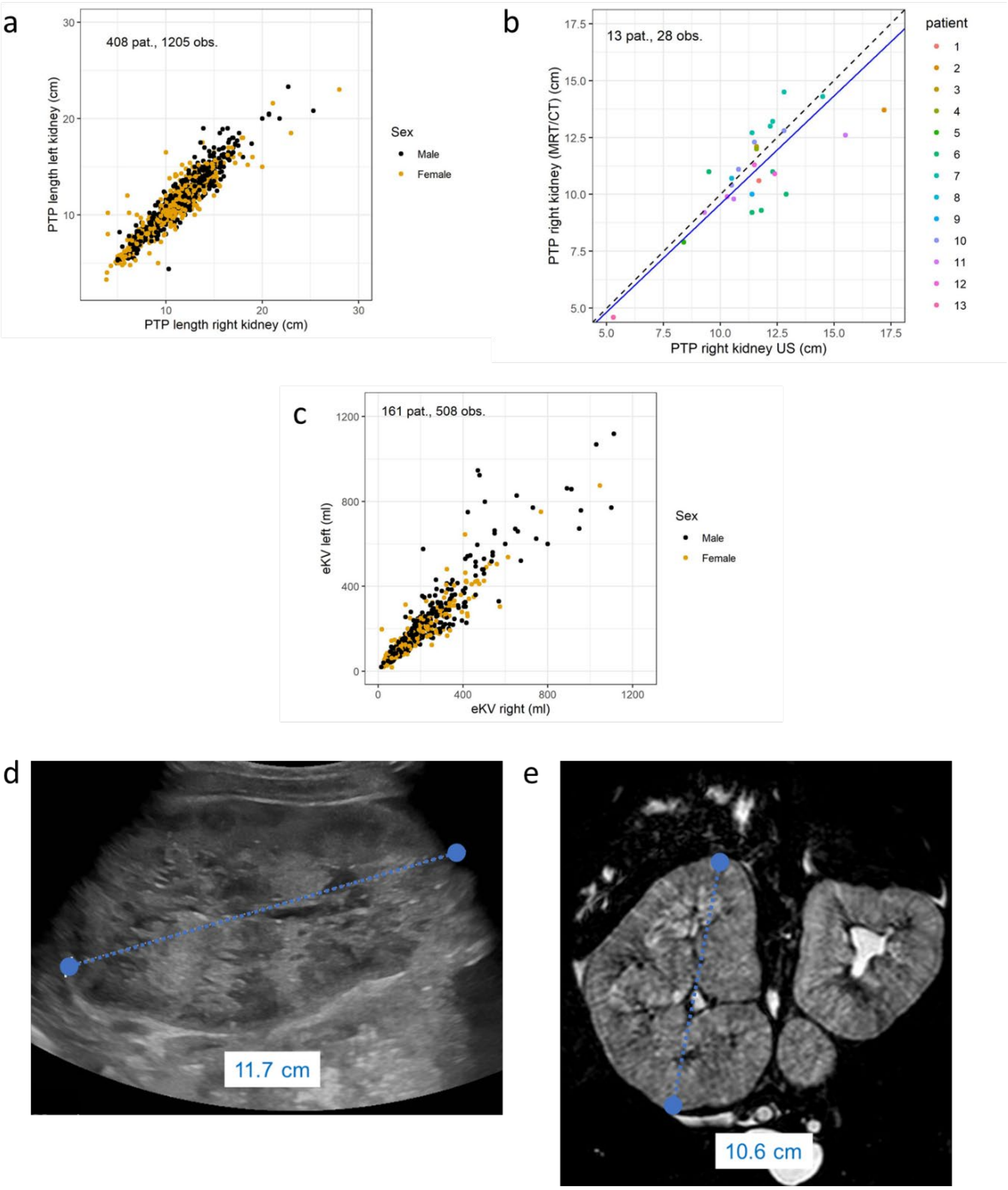

Supplementary Figure S1: Correlations of sonographic pole-to-pole length measurements between right and left kidney (a), of pole-to-pole length measurement based on CT or MRI versus ultrasound (b) and of sonographic kidney volume estimations between right and left kidney (c). Dashed line in (b) represents unbiased correlation, blue line represents results of orthogonal regression. Exemplary presentation of pole-to-pole length measurements of the right kidney based on ultrasound (d) and MRI (e) in a patient (patient #1 in b) at the age of 0.5 years, where ultrasound slightly overestimates kidney length.

Supplementary Figure S2:

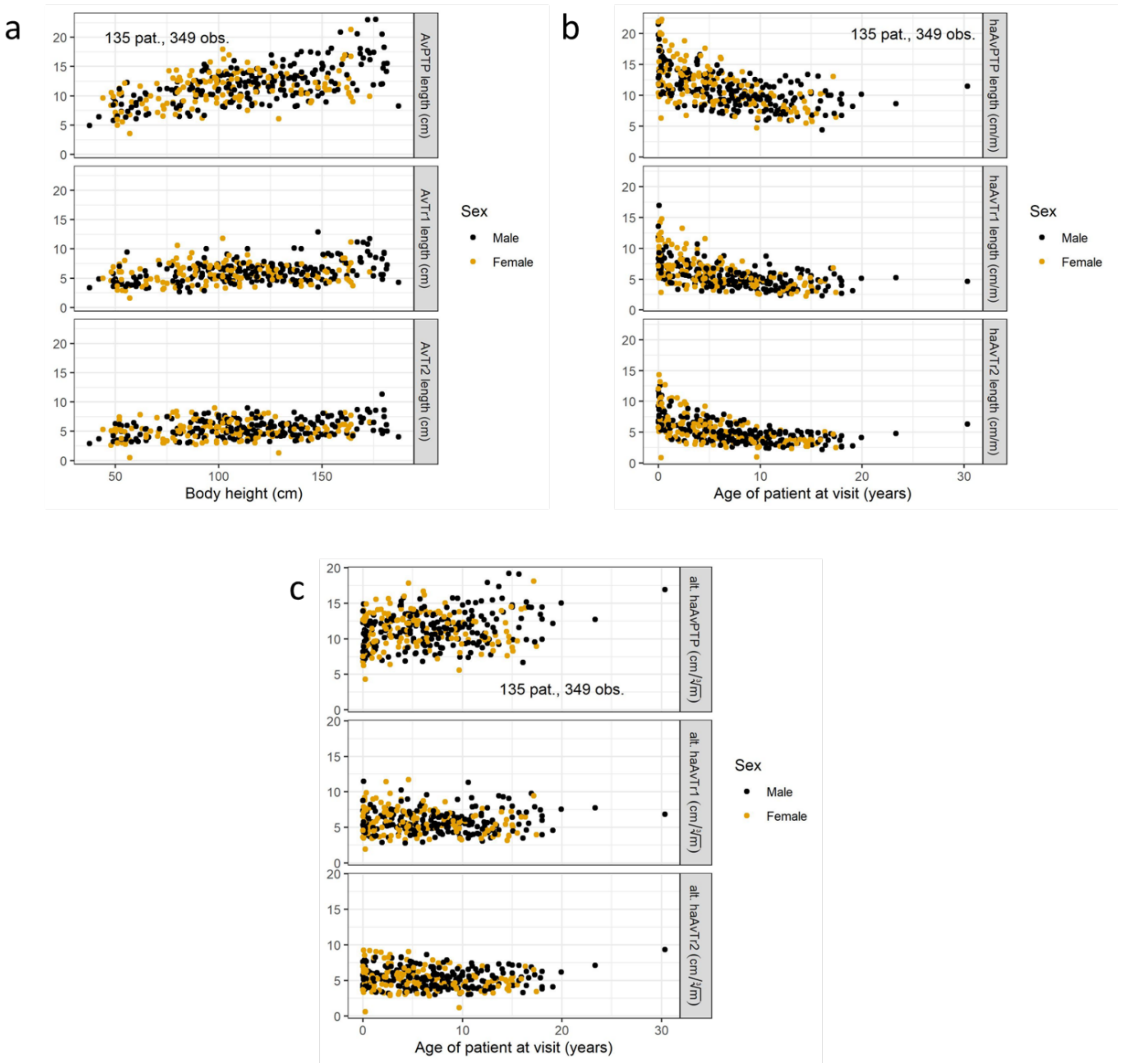

Supplementary Figure S2: Correlations of average pole-to-pole length and transverse lengths 1 and 2 to body height (a). Average pole-to-pole length is generally bigger than the other dimensions and the relation to body height is also stronger. However, also the other two dimensions grow with body height. Height adjusted length declines with age in every dimension (b). To investigate why this effect is not visible for height adjusted estimated TKV, we changed the height adjustment. Instead of dividing by body height, we divide by $\sqrt[3]{body\ height}$, to account for the different dimensionality between volume and length. We see that the decline vanishes in every dimension (c).

Supplementary Figure S3:

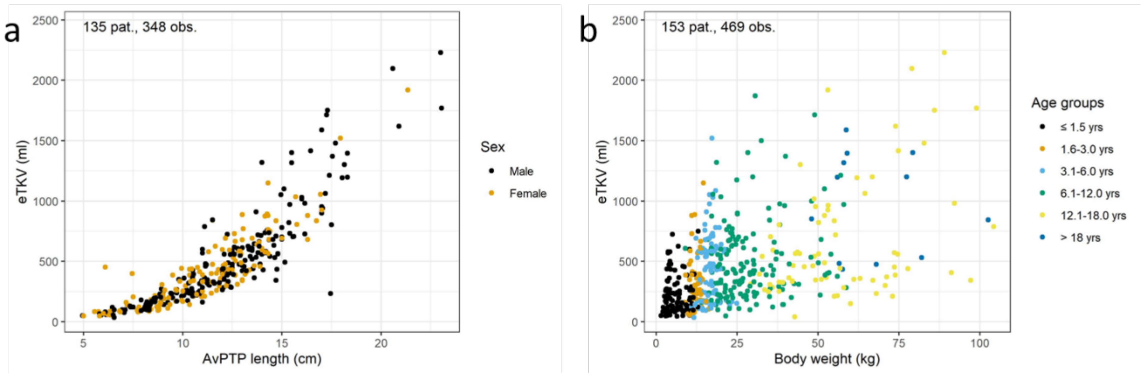

Supplementary Figure S3: Description of the correlations between average pole-to-pole length and estimated TKV (a), and between body weight in different age groups and estimated TKV (b).

Supplementary Figure S4:

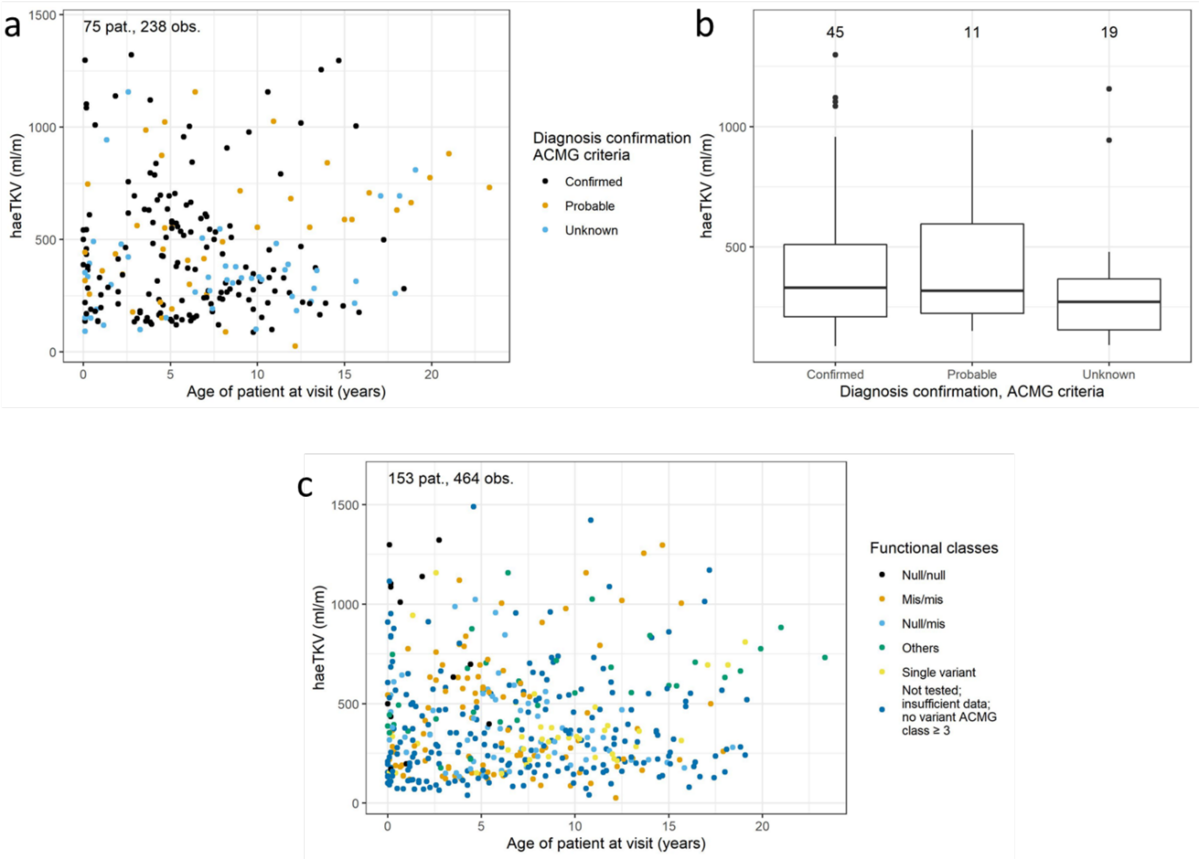

Supplementary Figure S4: Correlation of height-adjusted estimated TKV values and genetics: Development of height-adjusted estimated TKV values over time according to diagnosis confirmation (a), box-plots of first documented height-adjusted estimated TKV values for the subgroups of genetic confirmation of ARPKD (b), and development of height-adjusted estimated TKV values over time according to functional classes of *PKHD1* variants (c).

Supplementary Figure S5:

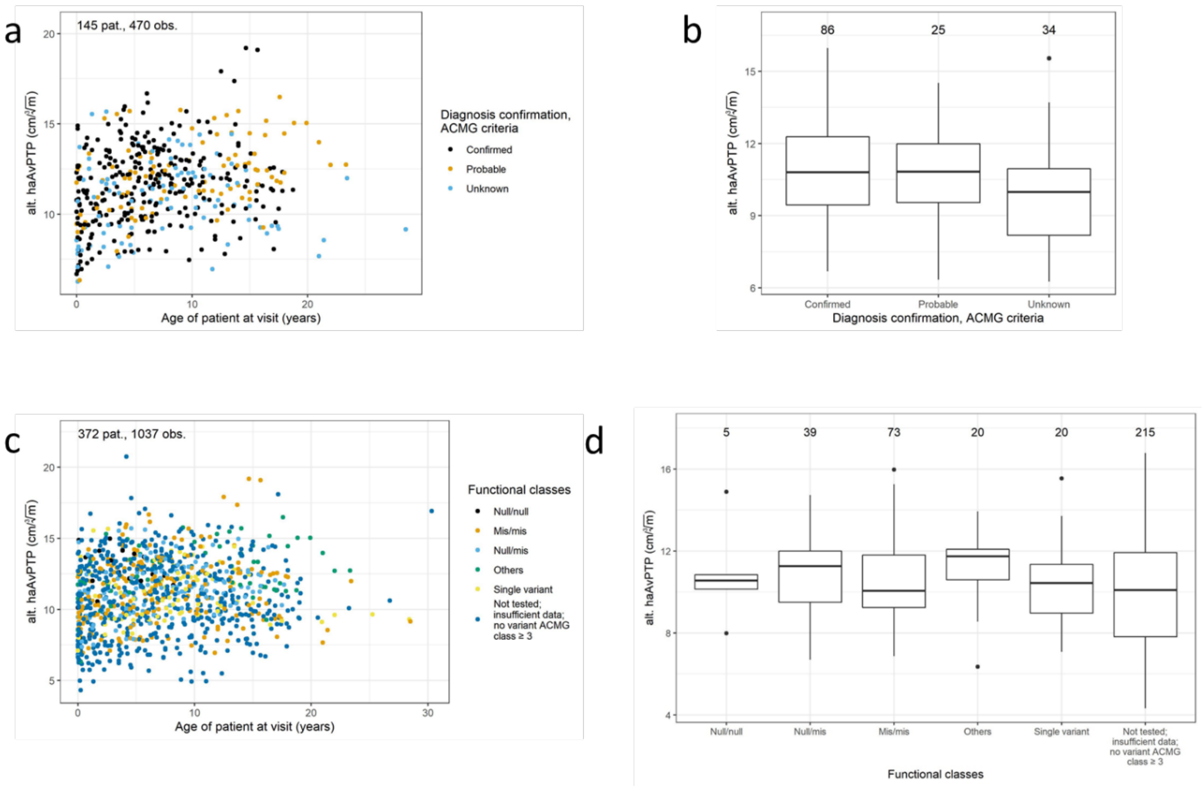

Supplementary Figure S5: Correlation of alternative height-adjusted PTP length values and genetic findings does not reveal differences between subgroups. Development of alternative height-adjusted PTP length values over time according to diagnosis confirmation (a), box-plots of first documented alternative height-adjusted PTP length values for the subgroups of genetic confirmation of ARPKD (b), development of alternative height-adjusted PTP length values over time according to functional classes of *PKHD1* variants (c), and box-plots of first documented alternative height-adjusted PTP length values for functional classes of *PKHD1* variants (d).

Supplementary Figure S6:

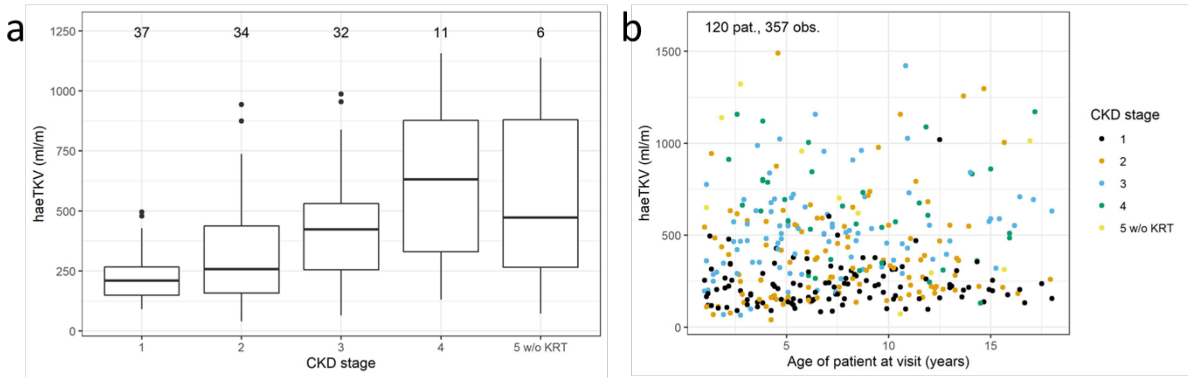

Supplementary Figure S6: Correlation of height-adjusted estimated TKV values and kidney function. For each patient only the first documented values beyond the first year of life have been used. Higher first documented height-adjusted estimated TKV values are seen with higher CKD stage G classes (a). There is a wide but stable distribution of height-adjusted estimated TKV values over time according to CKD stages (b).

Supplementary Figure S7:

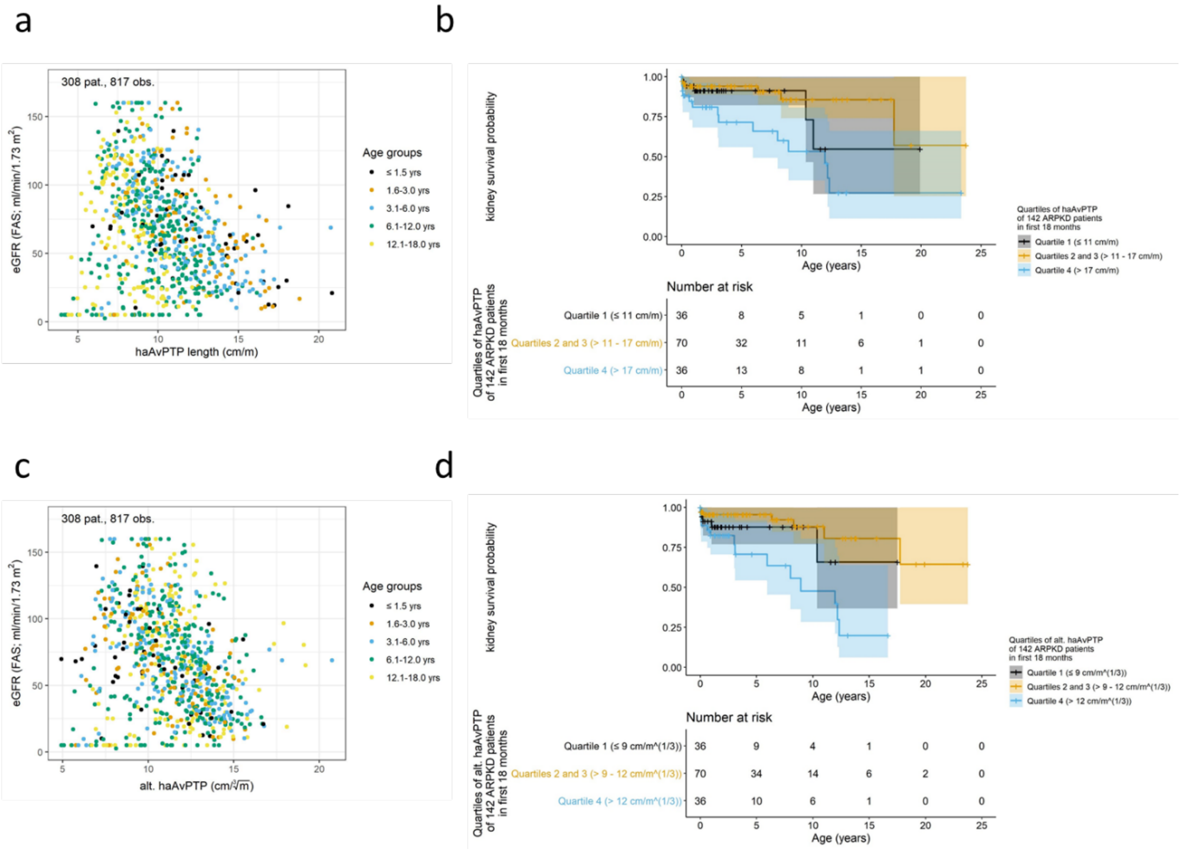

Supplementary Figure S7: Correlation of height-adjusted average PTP length and alternative height-adjusted average PTP length to kidney function and KRT-free kidney survival. Both measures show loose inverse correlation to eGFR (a, c). Stratification according to quartiles of the highest documented values in the first 18 months show a trend towards worst kidney survival in the highest quartile for both height-adjusted average PTP length and alternative height-adjusted average PTP length (b, d).

Supplementary Figure S8:

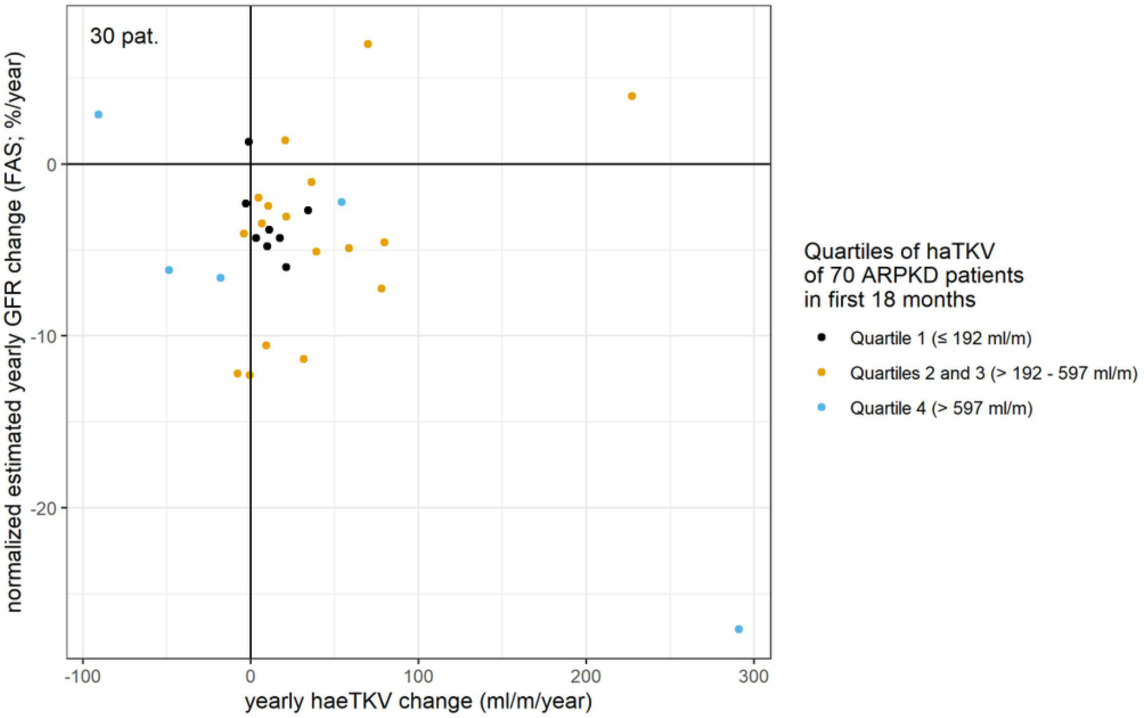

Supplementary Figure S8: Correlation of normalized yearly eGFR change and yearly haeTKV change stratified according to haeTKV quartiles.
